# Time-dependent improvement in functional outcome following LCS rotating platform knee replacement: A prospective longitudinal study in 50 patients

**DOI:** 10.3109/17453674.2010.533929

**Published:** 2010-11-26

**Authors:** Tor Kjetil Nerhus, Stig Heir, Elisabeth Thornes, Jan Erik Madsen, Arne Ekeland

**Affiliations:** ^1^Martina Hansens Hospital, Bærum; ^2^Orthopaedic Center, Oslo University Hospital, and Faculty of Medicine, University of Oslo, Oslo, Norway

## Abstract

**Background and purpose:**

Long-term follow-up studies after total knee replacement (TKR) using an LCS rotating platform have shown survival rates of up to 97%. Few studies have evaluated short-term functional outcome and its improvement over time. We determined the time course of functional outcome as evaluated by the knee injury and osteoarthritis outcome score (KOOS) over the first 4 years after TKR using the LCS mobile bearing.

**Patients and methods:**

50 unselected patients (mean age 70 (40–85) years, 33 women) with osteoarthritis in one knee underwent TKR with an LCS mobile bearing. Data were collected by an independent investigator preoperatively and at 6 weeks, 3 months, 6 months, 1 year, 2 years and 4 years postoperatively. KOOS, a self-assessment function score validated for this purpose, and range of motion (ROM) were determined at all follow-ups.

**Results:**

The mean KOOS pain score increased from 43 before surgery to 66 at 6 weeks and 88 at 2 years. It was 84 at 4 years. The mean KOOS activities of daily living score (ADL) increased from 49 before surgery to 73 at 6 weeks, then gradually to 90 at 2 years. It decreased to 79 at 4 years. Mean passive ROM was 112° before surgery, 78° at departure from hospital, and then gradually increased to 116° at 2 years and 113° at 4 years.

**Interpretation:**

Recovery after TKR is time-dependent. Most of the expected improvement in pain and function is achieved at 6 months postoperatively, but some further improvement can be expected up to 2 years postoperatively. ROM will also gradually improve up to 2 years after TKR, and reach the same level as before surgery.

Long-term follow-up studies after total knee replacement (TKR) using the cemented LCS rotating platform have shown survival rates of up to 97% at 15 and 20 years (Buechel 2002, Callaghan et al. 2005). Survival rates provide important information to doctors and patients, but from the patient's point of view, it is equally important to know how well their knee will function with a TKR, both in the short term and the long term.

Recent studies focusing on patient-relevant functional outcome after TKR have provided some information about recovery after surgery. Lingard et al. (2004) found that patients with co-morbid conditions preoperatively were more likely to have an inferior outcome at 1 and 2 years after TKR. Fitzgerald et al. (2004) analyzed quality of life (QoL) outcome before—and the first year after—TKR, and found that physical function was worse after 1 month and then improved. Kennedy et al. (2006) studied the early postoperative period (4 months) after TKR, and established a timeline for recovery using Western Ontario and McMaster Universities osteoarthritis index subscales (WOMAC). They found that preoperative function and gender (with men having better results than women) were predictive of functional recovery. In a later study, Kennedy et al. (2008) used the lower extremity functional scale to study recovery during the first year after TKR. They found that the greatest improvement occurred over the first 3 months, and that little improvement occurred after 6 months. The KAT Trial Group (2009) evaluated the effects of metal backing, patellar resurfacing, and mobile bearing using the Oxford knee score. They found no evidence of an effect of these variants on functional recovery up to 2 years after TKR. Nilsdotter et al. (2009) used the knee injury and osteoarthritis outcome score (KOOS) to study improvement in pain and physical function during the first 5 years after TKR, and found that although a significant improvement was seen 5 years postoperatively, the best result was reported at 1 year. Most of these studies had follow-up times of less than 1–2 years; only one had a longer follow-up (Nilsdotter et al. 2009).

In this prospective study, we determined (1) the time course of patient-relevant functional outcome as evaluated by KOOS, and (2) the time course of range of motion (ROM) during the first 4 years after TKR using the LCS rotating platform prosthesis. Improvement in patient self-reported pain and daily function during the study period were of particular interest.

## Patients and methods

50 consecutive patients operated with total knee arthroplasty at Martina Hansens Hospital between February and September 2003 were included in a prospective observational, hypothesis-generating study. All patients were operated with the cemented Low Contact Stress (LCS) rotating platform prosthesis (DePuy, Warsaw, IN). This implant had been used at our hospital since 1994. The inclusion criteria were: patients with knee osteoarthritis (no rheumatoid arthritis or previous knee infection), aged 40-85 years, who had been admitted for total knee replacement. Informed consent was obtained from all patients. Data were collected by an independent investigator (a physiotherapist) preoperatively and at 6 weeks, 3 months, 6 months, 1 year, 2 years, and 4 years postoperatively.

### Knee injury and osteoarthritis outcome score (KOOS)

Patients completed of the KOOS (Roos et al. 1998), Norwegian version, later published by Lygre and Furnes (2007), on their own at each follow-up. The KOOS is a 42-item self-administered questionnaire based on the WOMAC osteoarthritis index, which has been proven to be valid for subjects with total knee replacement (Roos and Toksvig-Larsen 2003). The KOOS has 5 subscales: pain (9 items), other symptoms (7 items), activities of daily living (ADL) (17 items), sports and recreational function (sport/rec) (5 items), and knee-related quality of life (QoL) (4 items). A score from 0 to 100 is calculated for each subscale, with 100 representing the best result. The following question was added to the KOOS form: “Does any other illness affect your function in daily activities to a higher degree than your knee?”.

### Range of motion

Non-weight-bearing active and passive ROM values were obtained with the patient in the supine position in order to allow free hip flexion. A goniometer was used (Norkin et al. 1996).

### Complications

Patients were asked about complications at all follow-ups.

### Operative technique

The operation was performed under spinal/epidural anesthesia using a midline skin incision 12–15 cm in length, followed by a medial parapatellar approach. Approximately 10 mm of the tibial plateau was resected, aiming at a posterior slope of 7˚. The distal part of the femoral condyles was resected, attempting to achieve femorotibial alignment of 5˚ of valgus in the coronal plane. Distal and posterior femoral condylar resection was performed to remove a volume of bone that matched the size of the femoral component to be implanted. If needed, ligament and/or capsular release was performed to balance the flexion and extension gaps and to overcome any flexion contracture. The patella was not resurfaced. All implants were inserted with Palacos cement after pulsed lavage. All patients began walking with a frame, bearing full weight and starting range of movement (ROM) exercises on the first postoperative day.

### Statistics

The primary outcome measures were the KOOS subscales for pain and for ADL. These subscales were chosen as primary outcome measures because they were thought to be most important for our patients. The secondary outcome measures were the KOOS subscales for symptoms, QoL, sport/rec, and the measurements of active and passive ROM. Statistical analysis was performed using SPSS for Macintosh version 18. The data were analyzed by fitting separate linear mixed models for the KOOS subscales, and for the ROM measurements, choosing an unstructured (i.e. completely general) covariance matrix for the repeated measurements. Missing data were assumed to be missing at random. The mean of each outcome was estimated along with its corresponding 95% confidence interval (CI) at all observation times. Post-hoc comparisons between the main effects of all pairs of points in time (that is, no reference category defined) were performed separately for each model, corresponding to the KOOS subscales and active and passive ROM. Bonferroni adjustments, including all pairwise comparisons within a specific model, were applied to the respective confidence intervals and p-values to account for multiple testing. The significance level was set at 0.05, and all differences with p-values below this level were considered to be statistically significant.

## Results

All 50 patients originally included in the study were included in the statistical analyses: 33 women and 17 men, with a mean age of 70 (40–85) years. 24 left knees and 26 right knees were operated by 6 surgeons, with a mean operating time of 76 (54–150) min.

### Missing data

A total of 350 evaluations were planned for the study population of 50 patients. 33 of these evaluations (9%) were missing in 11 patients. 9 patients failed to attend one or more of the postoperative evaluations and 2 patients died between the 2- and 4-year evaluations. These 11 patients were compared to the 39 patients with complete data regarding preoperative differences. The mean age of the patients with missing data was 75 years, and it was 68 years for the group with complete data. 4 of the 11 patients with missing data were males, as compared to 13 of the 39 in the complete data group. The preoperative KOOS values were similar. Mean preoperative KOOS subscale pain score was 42 in the missing data group and 44 in the complete data group. Mean preoperative KOOS subscale ADL was 48 in the missing data group and 49 in the complete data group.

### Complications

Complications related to the surgery were: ruptured patellar tendon (1 patient), deep infection (1), partial dropfoot (1), arthrofibrosis (1), and urinary tract infection (1). Complications related to the spinal/epidural anesthesia were: persistent spinal root pain (1) and meningitis (1). Most complications were transient. The ruptured patellar tendon was repaired with a good result. The arthrofibrosis was operated with open release 1 year postoperatively, with a good result. The meningitis was treated with antibiotics and healed without sequelae. The spinal root pain, however, became a chronic problem.

### Knee injury and osteoarthritis outcome score (KOOS)

The linear mixed models analysis revealed that there were statistically significant differences in KOOS scoring between the 7 times of measurement. This was true for all subscales of the KOOS (p < 0.001).

**Figure 1.**
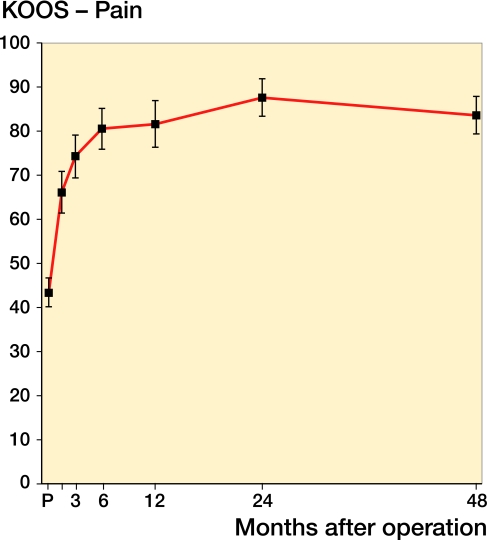
Graph showing improvement in the KOOS pain subscale with time. Values are mean ± CI. Pairwise comparisons revealed statistically significant improvement between P and all other time points (p<0.001), and between 3 months and 2 years (p = 0.02). P: preoperatively.

Mean KOOS score values for pain continued to improve until 2 years after surgery, then declined towards 4 years. Only small changes were seen after 6 months (Figure 1). Using the 2-year scoring values as a reference, the percentages of improvement in KOOS pain achieved were 51% at 6 weeks, 84% at 6 months, 87% at 1 year, 100% at 2 years, and 91% at 4 years.

**Figure 2.**
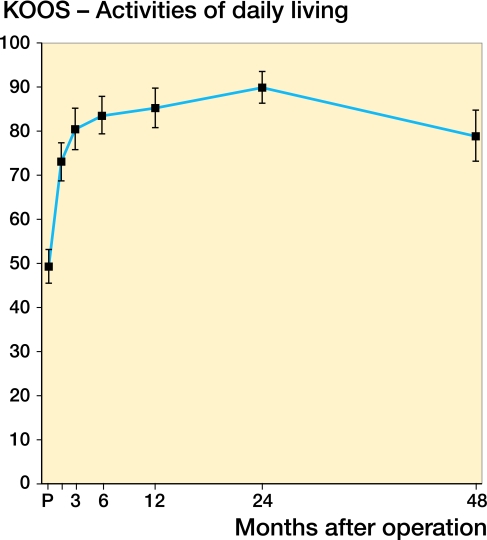
Graph showing improvement in the KOOS ADL subscale with time. Values are mean ± CI. Pairwise comparisons revealed statistically significant improvement between values preoperatively and at all other time points (p < 0.001) and between those at 6 months and 2 years (p = 0.005). Values at 4 years were significantly worse than at 2 years (p < 0.001). See legend to Figure 1 for explanation of abbreviations.

Mean KOOS score values for ADL continued to improve until 2 years after surgery, and declined again towards 4 years. Only small changes were observed after 6 months (Figure 2). Using the 2-year scoring values as a reference, the percentages of improvement in KOOS ADL achieved were 59% at 6 weeks, 84% at 6 months, 89% at 1 year, 100% at 2 years, and 73% at 4 years.

**Figure 3.**
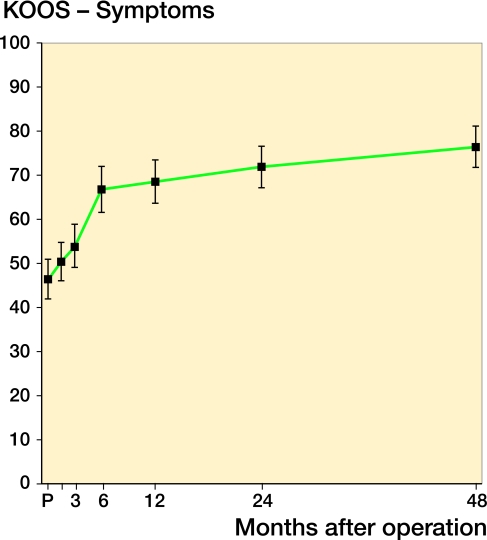
Graph showing improvement in the KOOS symptoms subscale with time. Values are mean ± CI. Pairwise comparisons revealed statistically significant improvement between values preoperatively and at 6m (p < 0.001), between values preoperatively and at 1 year (p < 0.001), between values preoperatively and at 2 years (p < 0.001), between values preoperatively and at 4 years (p < 0.001), and between values at 6 months and 4 years.

Mean KOOS score values for symptoms continued to improve at all time points until 4 years after surgery (Figure 3). Using the 4-year scoring values as a reference, the percentages of improvement in KOOS symptoms achieved were 13% at 6 weeks, 68% at 6 months, 74% at 1 year, 85% at 2 years, and 100% at 4 years.

**Figure 4.**
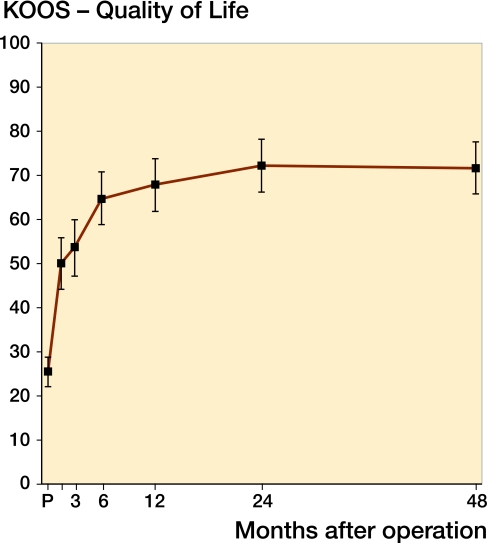
Graph showing improvement in the KOOS QoL subscale with time. Values are mean ± CI. Pairwise comparisons revealed statistically significant improvement between values preoperatively and all other time points (p < 0.001), between values at 3 months and 1 year (p = 0.02), between values at 3 months and 2 years (p < 0.001), and between values at 3 months and 4 years (p < 0.001).

Mean KOOS score values for QoL continued to improve until 2 years after surgery (Figure 4). Using the 2-year scoring values as a reference, the percentages of improvement in KOOS QoL achieved were 53% at 6 weeks, 84% at 6 months, 91% at 1 year, 100% at 2 years, and 99% at 4 years.

**Figure 5.**
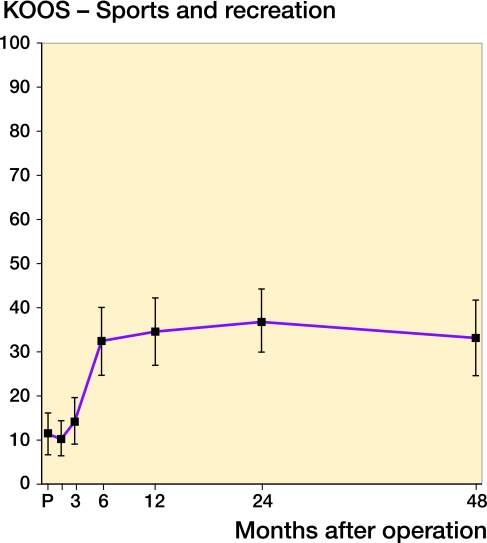
Graph showing improvement in the KOOS sport/rec subscale with time. Values are mean ± CI. Pairwise comparisons revealed statistically significant improvement between values preoperatively and at 6 months (p < 0.001), between values preoperatively and at 1 year (p < 0.001), between values preoperatively and at 2 years (p < 0.001), between values preoperatively and at 4 years (p < 0.001), and between values at 3 months and 6 months (p < 0.001).

Mean KOOS score values for sport/rec continued to improve until 2 years after surgery, but minimal changes were seen after 6 months (Figure 5). Using the 2-year scoring values as a reference, the percentages of improvement in KOOS sport/rec achieved were 0% at 6 weeks, 83% at 6 months, 92% at 1 year, 100% at 2 years, and 86% at 4 years.

At the 4-year evaluation, 39 patients responded to the supplementary question: “does any other illness affect your function in daily activities to a higher degree than your knee?”. 20 patients reported having such an illness and 19 patients did not. The first group had a decrease of 9 points and the second group had a decrease of 10 points in KOOS ADL from 2 years to 4 years.

### Range of motion

**Figure 6.**
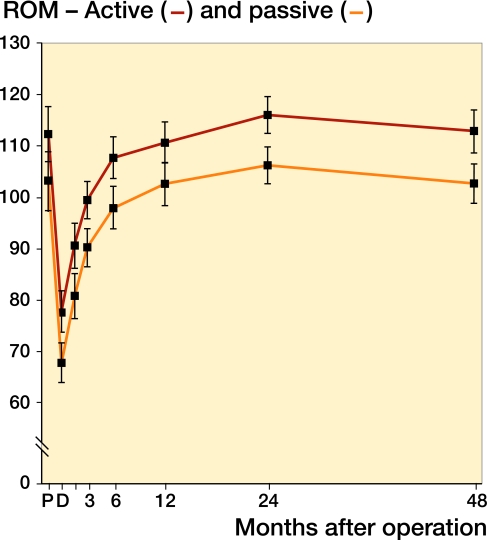
Graph showing improvement in active ROM (red line) and passive ROM (blue line) with time. Values are mean ± CI. Pairwise comparisons for active ROM revealed statistically significant differences between values at discharge from hospital and at all other time points (p < 0.001), between values at 6 months and 1 year (p = 0.004), between values at 6 months and 2 years (p < 0.001), and between values at 6 months and 2 years (p = 0.001). Pairwise comparisons for passive ROM revealed statistically significant differences between values at discharge from hospital and at all other time points (p < 0.001), between values at 6 months and 2 years (p < 0.001), and between values at 1 year and 2 years (p = 0.005). P: preoperatively; D: discharge from hospital.

The linear mixed models analysis revealed statistically significant differences in both active and passive ROM between the 8 times of measurement (p < 0.001). Active and passive ROM mean score values continued to improve until 2 years after surgery, and declined again towards 4 years (Figure 6). Using the 2-year ROM values as a reference, the percentages of improvement in passive ROM were 34% at 6 weeks, 78% at 6 months, 86% at 1 year, 100% at 2 years, and 91% at 4 years.

Patients with complete data evaluated at 2 years (n = 41) were divided into groups according to their preoperative ROM. Patients with a preoperative ROM of 80–99 degrees (n = 7) improved their ROM (from 87 to 112 degrees). Patients with a preoperative ROM of 100–119 degrees (n = 14) ended up with approximately the same ROM (from 111 to 113 degrees), whereas patients with a ROM of 120–160 degrees (n = 20) preoperatively had a somewhat worse ROM (from 128 to 121 degrees) 2 years postoperatively.

### Discussion

Historically, functional outcome after TKR has been based on objective measurements on physical examination, such as range of motion, ligamentous laxity, performance on functional tests, and radiographic findings. Frequently used older scoring systems, such as the hospital for special surgery score (Insall et al. 1976) and the Knee Society score (Insall et al. 1989) rely on the examiner to ask questions and perform tests. They include questions that measure pain, range of motion, stability, alignment, and function. These scores use objective values that are useful to surgeons, but they do not take into account the patient's satisfaction regarding health and function. During the last decade, several authors have emphasized the importance of measuring the patient's own experience of disability using self-reported questionnaires (Marx 2003, Garratt et al. 2004, Tanner et al. 2007). Different scales that measure specific health considerations such as function during daily activities and pain have been developed. In a study comparing whether different knee-specific outcome instruments included items to detect symptoms and disabilities most important to the patients, the KOOS (Roos et al. 1998, Roos and Toksvig-Larsen 2003) and the International Knee Documentation Committee standard evaluation form (IKDC) were identified as the top two general knee quality-of-life instruments ensuring that the patient's point of view was considered (Tanner et al. 2007). We used the Norwegian version of the KOOS, later published by Lygre and Furnes (2007). KOOS pain and KOOS ADL were chosen as primary outcome measures, as we assumed that they would be most relevant to the patients under study.

Other authors (Kennedy et al. 2006, 2008) have included more objective parameters such as the 6-minute walk test, the timed up-and-go test, and the timed stair test. In our study, ROM was the only objective parameter studied. Preferably, a study of functional results after TKR should include both a patient-relevant self-evaluation score and more demanding objective tests. Such tests would obviously have strengthened our study, but they were not included because of limited resources.

Our study has other limitations as well. One weakness is the amount of missing data. When designing the study, we decided to use linear mixed models statistics. This method allows some missing data; thus, all the patients included could still remain in the study. The missing data analysis, however, revealed small differences between patients with missing data and patients with complete data. Another weakness was the method of measuring ROM: clinically with a goniometer. Ryd et al. (1997) found that clinical measurements of, for example, flexion and extension have questionable reliability. Lenssen et al. (2007) showed that reliability of clinical ROM measurement was acceptable with regard to group comparisons but poor with regard to individual measurements over time. Measurements on radiographs may be a more reliable way to examine ROM (Lavernia et al. 2008), but they are more expensive and time consuming.

We found substantial improvement in pain and function as early as 6 weeks postoperatively; approximately 50% of the improvement in KOOS ADL and KOOS pain were achieved by 6 weeks. One of the strengths of our study was that we included both short-term and intermediate-term evaluations, which were necessary to establish a true timeline for functional recovery. Other studies have focused on either short-term (Kennedy et al. 2006, 2008) or long-term evaluations (Nilsdotter et al. 2009).

Our findings suggests that improvement in KOOS ADL and KOOS pain scores continues until 2 years postoperatively. This is in contrast to the study of Nilsdotter et al. (2009), who obtained the best result at 1 year after TKR, but without collecting data between 1 and 5 years. In our study, the improvements in KOOS pain and ADL scores were not statistically significant between all time points, and the differences were fairly small after 6 months. Whether this improvement between 6 months and 2 years is clinically significant is therefore debatable. The smallest detectable clinical improvement in KOOS pain and ADL scores is suggested to be 8–10 score units (Roos and Toksvig-Larsen 2003). Mean KOOS pain score rose from 81 at 6 months to 88 at 2 years. Mean KOOS ADL score rose from 85 at 6 months to 90 at 2 years. The difference between the 6-month results and the 2-year results was thus smaller, but close to the proposed smallest detectable clinical improvement. In terms of patient information, it would be fair to say that most of the improvement in pain and ADL scores after TKR is achieved at 6 months, but that some further improvement can be expected up to 2 years postoperatively.

We found that the KOOS ADL score was lower at 4 years than at 2 years, decreasing from 90 to 79 points. This coincides with the findings of Nilsdotter et al. (2009); their result for KOOS ADL was worse at 5 years than at 1 year. No predictors of postoperative physical function were found in their study, indicating the difficulty in determining preoperatively who will benefit more (or less) from the procedure. The ADL subscore is the subscore of KOOS most likely to be influenced by factors other than the patient's knee. Thus, in our study, we included the following question at the 4-year evaluation: “Does any other illness affect your function in daily activities to a higher degree than your knee?”. 20 patients stated that they had such an illness and 19 patients did not. Both groups, however, had a similar decrease in KOOS ADL from 2 years to 4 years.

Paradowski et al. (2006) have collected population-based reference data for the KOOS in different age groups. Reference data for KOOS ADL in the age group 55–74 years was 86 for men and 77 for women. In the age group 75–84 years, it was 76 for men and 83 for women. This indicates that our results at 2 years (90) were somewhat better than in the reference population, and that our 4-year results (79) were close to the reference population data. The reason for the inferior results at 4 years compared to the 2-year follow-up is uncertain. The placebo effect influences results after orthopedic surgery (Moseley et al. 2002, Hróbjartsson and Gøtzsche 2004, Kirkley et al. 2008). Thus, the patients may score better at an earlier time when they are more enthusiastic about the procedure, whereas after 4 years they score more “realistically”. Further research may enlighten us on this matter.

Earlier studies have indicated that the primary factor determining postoperative ROM is the preoperative ROM (Ritter et al. 2003, Dennis et al. 2007). Our study confirmed these findings, showing similar mean passive ROM preoperatively (112 degrees) and at 4 years postoperatively (114 degrees). Furthermore, when the patients were divided into subgroups according to their preoperative ROM, the results showed some interesting differences; the stiffer knees (80–99 degrees) gained range of motion, while the more mobile knees (100–119 degrees) kept their ROM unchanged, and the most mobile knees preoperatively (120–160 degrees) appeared to lose some of their ROM.

In summary, functional results and recovery after TKR are time-dependent. Substantial improvements in pain and function were present as early as 6 weeks postoperatively, and most of the expected improvements had been achieved by 6 months. Some further improvement was, however, observed up to 2 years postoperatively, before a slight decline in function occured towards 4 years. ROM gradually improved up to 2 years after TKR, to the same level as prior to surgery. Between 2 and 4 years a slight decline in ROM was observed, paralleling the KOOS scores.
